# Aeroelastics-aware compensation system for soft aerial vehicle stabilization

**DOI:** 10.3389/frobt.2022.1005620

**Published:** 2022-11-09

**Authors:** Fernando Ruiz, Begoña C. Arrue, Aníbal Ollero

**Affiliations:** GRVC Aerial Robotics Laboratory, University of Seville, Seville, Spain

**Keywords:** soft robotics, UAVs, soft actuation, aerodynamics, multirotor dynamics

## Abstract

This paper describes a compensation system for soft aerial vehicle stabilization. Balancing the arms is one of the main challenges of soft UAVs since the propeller is freely tilting together with the flexible arm. In comparison with previous designs, in which the autopilot was adjusted to deal with these imbalances with no extra actuation, this work introduces a soft tendon-actuated system to achieve in-flight stabilization in an energy-efficient way. The controller is specifically designed for disturbance rejection of aeroelastic perturbations using the Ziegler-Nichols method, depending on the flight mode and material properties. This aerodynamics-aware compensation system allows to further bridge the gap between soft and aerial robotics, leading to an increase in the flexibility of the UAV, and the ability to deal with changes in material properties, increasing the useful life of the drone. In energetic terms, the novel system is 15–30% more efficient, and is the basis for future applications such as object grasping.

## 1 Introduction

The use of unmanned aerial vehicles (UAVs) has grown exponentially in recent years (Rao et al., 2016). It has been demonstrated that this technology offers a cost-effective solution to several operations such as surveillance, monitoring and inspection (Bernard et al., 2011; Ruggiero et al., 2018). Many of these applications require physical interaction with the environment; for instance, autonomous construction (Augugliaro et al., 2014), contact-based inspection (Tognon et al., 2019), package delivery (Grzybowski et al., 2020), or manipulation (Jimenez-Cano et al., 2013). Multirotors are particularly suitable for these tasks, as they can hover in-place and present Vertical Take-Off and Landing (VTOL) capabilities.

Unfortunately, traditional UAVs have rigid structures that limit their ability to interact with the environment, especially humans. In recent years, the field of soft aerial robotics (Rus and Tolley, 2015) has benefitted from the utilization of reconfigurable, flexible, soft, and morphologically adaptive structures (Mintchev et al., 2018; Riviere et al., 2018; Dilaveroğlu and Özcan, 2020). The introduction of these concepts in aerial robots leads to improved maneuverability and efficiency (Ajanic et al., 2020); multi-modal mobility across terrain interfaces and fluid boundaries (Zufferey et al., 2019); robustness to landing and collision (Ruiz et al., 2022a); manipulation and perching (Garcia Rubiales et al., 2021; Ruiz et al., 2022b); bio-inspired aerial construction (Dufour et al., 2016; Kornatowski et al., 2017; Yang et al., 2019).

UAV systems can become even more efficient if they work in cooperation with human beings (ZHAO et al., 2020; Zheng et al., 2021). The propulsion system is one of the main challenges to solve in order to perform these tasks safely and efficiently, hence deformable propellers have become a real alternative (Nguyen et al., 2020). In the same way, robotic arms can employ soft-end effectors for contact manipulation (Xiang et al., 2019; Zhang et al., 2019). Soft actuators can provide a greater force-weight ratio than traditional servos (Gomez-Tamm et al., 2019).

Many of the soft aerial robot designs are based on animals and try to mimic their behavior (Kovac, 2016; Sareh et al., 2017). However, the control of these naturally complex and non-linear soft robots is an arduous task, since flexibility increases significantly the mathematical complexities in the modeling when comparing to rigid robots (Fishman and Carlone, 2020). Furthermore, these models can quickly become outdated with changes in material properties during the life cycle. This is the reason why some authors have switched to data-based approaches (Kim et al., 2021).

A great research effort is being spent on the field of adaptive control techniques (Rowe, 2009; Benosman, 2016). This approach can become extremely useful to cope with unknown or time varying dynamics. Unfortunately, the development of this type of control usually remains in mere theoretical studies, and they are rarely introduced in the industry due to their complexity and computational load. Therefore, these techniques have been simplified to give rise to simple PID architectures with autotuning capabilities (Leva, 1997; Leva, 2005; Sahputro et al., 2017).

This article presents a soft tendon-actuated compensation system, which is validated and integrated into a flexible aerial vehicle developed by the authors in previous works (Ruiz et al., 2022a; Ruiz et al., 2022b). Balancing the arms is one of the main challenges of soft UAVs since the propeller is freely tilting together with the flexible arm. In these previous works, modifications were made to the autopilot mixer to deal with these deflections of the arms and achieve stable flight, although there were limitations regarding the degree of flexibility, as well as the consequent efficiency loss. The system proposed in this paper allows to increase the efficiency of the soft vehicle at higher flexibilities.

This work shows a detailed analysis of the aeroelastic disturbances (aerodynamic interferences between the arms, bending and torsion of the structure, material degradation) presented by the soft UAV. The interpretation and modeling of these is fundamental for the design of a controller that adapts to changes in the response and to the different types of disturbance. The proposed controller is an adaptive PID designed using the Ziegler-Nichols method to respond optimally to changes in material properties or the appearance of aeroelastic effects during flight maneuvers.

The paper is structured as follows: Section 2 justifies the compensation system concept presented to solve the stabilization problem, and details the mechanical design of the system. Section 3 analyzes the aeroelastic disturbances it suffers. Section 4 deals with the design of the PID controller for each of the flight maneuvers depending on the type of disturbance. Section 5 evaluates the improvements in terms of energy efficiency of the system and its potential application to tasks such as grasping. Conclusions and new lines of research are proposed in Section 6.

## 2 System description

### 2.1 Problem statement

The use of flexible UAVs has great potential for interaction with the environment. Due to their flexible nature, they are much safer in the event of a collision. On the other hand, this flexibility can be used favorably in multiple applications: full-body perching on pipelines, trees and irregularly shaped objects; grasping of objects with their own arms without the need for an auxiliary system, with the consequent weight reduction; morphing in flight to access complex areas.

However, the widespread use of this kind of vehicles presents several challenges, mainly from the control point of view, specifically the stabilization of the arms since the propeller is freely tilting together with the flexible arm. For this reason, the need to implement an actuation system that controls these deflections is clear. The implementation of such a system is in turn quite challenging, given the complexity of obtaining a dynamic analytical model of a flexible and strongly non-linear system; and also, due to the continuous changes in material properties after successive cycles.

These uncontrolled arm deflections are a huge source of inefficiencies in the UAV, which are influenced by the aeroelastic disturbances suffered by the arm: aerodynamic interferences between the arms, bending and torsion of the structure, and material degradation. This work aims to provide insight into these aeroelastic effects and model the behavior of these disturbances as a function of the main parameters of the system.

Due to the complexity of these behaviors given the elasticity of the material, the models have been obtained from experimental results. For this, polynomial adjustments of first (k = 1) or second order (k = 2) have been performed based on the observed behavior, using Matlab.

In order to improve efficiency and increase flexibility, the PID controller proposed in this work must be specifically designed for disturbance rejection and self-adjust depending on the type of aeroelastic disturbance to which it is subjected according to the flight maneuver. In addition, a study of the limit angles at which the UAV can fly is carried out, which will serve as the basis for future grasping tasks.

### 2.2 Soft actuation system design

The mechanical design of the flexible arm has been studied in (Ruiz et al., 2022a) and the design of the UAV was detailed in (Ruiz et al., 2022b). This work uses the same actuation system through flexible tendons actuated by a servomotor, which allowed landing on pipes (perching maneuver). The novelty of this work lies in the extension of the use of this system during flight, in such a way that it is used as a compensation system for vehicle stabilization, increasing efficiency and flexibility of the prototypes.

**FIGURE 1:**
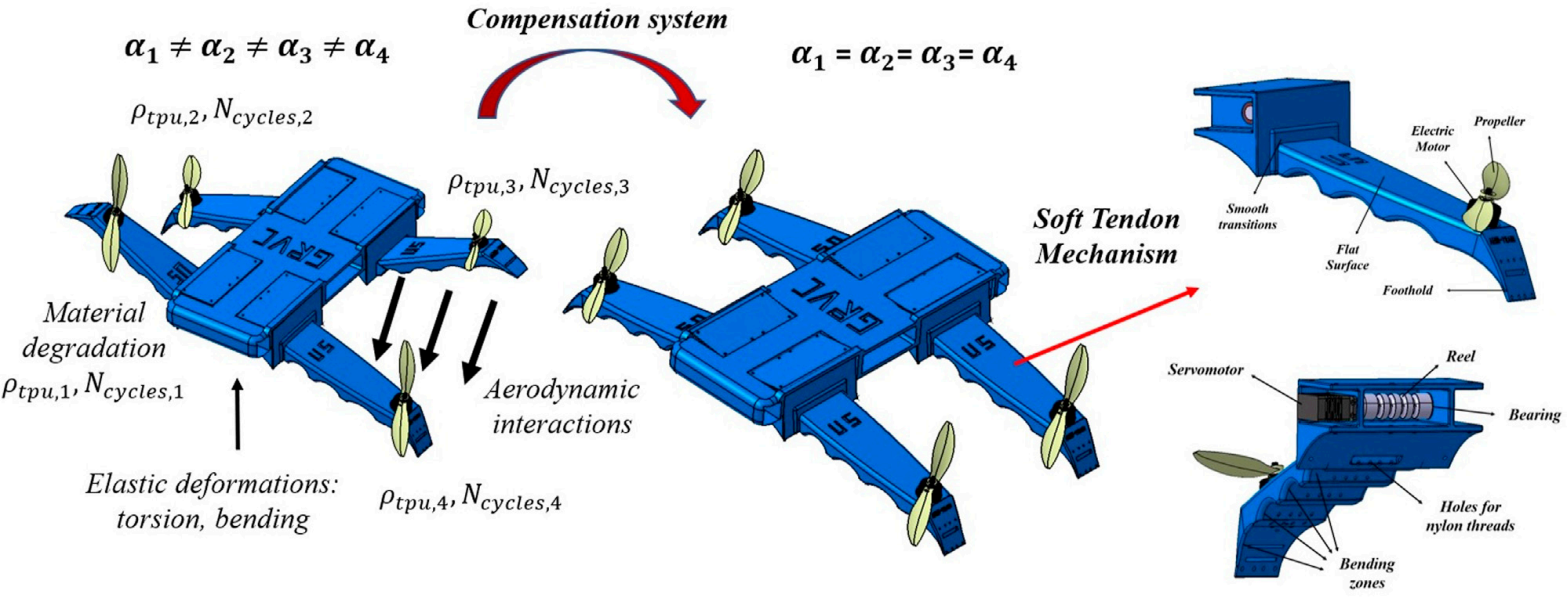
Novel concept for soft aerial vehicle stabilization: compensation system of aeroelastic perturbations (aerodynamics, bending, torsion, and changes in material properties) through soft tendons.

The equipment is the same as in previous works, composed of an electric motor (DJI 2312E, providing 450g of nominal thrust using a 4S (14.8V) battery), an electronic speed controller (DJI 430 Lite ESC) and a 10-inch DJI plastic propeller. The arm is equipped with soft tendons on its underside, responsible for generating compression forces that cause the arm to bend downwards (see Figure 1). These tendons are composed of nylon threads that are wound on a 3D-printed reel actuated by a HITEC MG996R servomotor which provides a maximum torque of 35 kgcm.

However, unlike (Ruiz et al., 2022a), in this work the control of the tendons (through the servomotors) in closed loop is not carried out through an FSR contact force sensor, but directly through an inertial measurement unit (IMU BNo055), which allows controlling the deflection angle of the arm.

For this reason, this work is divided into two fundamental parts that allow us to pursue the objective of stabilizing the arms in flight: modeling and analysis of the disturbances (aerodynamic and elastic) for each flight mode, analysis of the response of the system to these disturbances, and optimal design of the controller depending on the type of perturbation.

## 3 Analysis of aeroelastic perturbations

### 3.1 Aerodynamics

The aerodynamic interaction between the fluid flow of different rotors produces inefficiencies in the UAV, which is a widely studied problem that has led to standardized design criteria regarding the distances that must be left between them. In the flexible case, these interactions can lead to a destabilization of the UAV due to unexpected arm deflections generated by these changes in thrust. This section aims to particularize these studies to the case of flexible arms in which the relative angle between them is variable.

The natural way to model this behavior is through computational fluid dynamics (CFD), whose results must be validated through experimental tests, at least for a relevant number of cases. Numerical simulations have been performed using the commercial software Ansys Fluent. The Multiple Reference Frame (MRF) method is a steady state approximation suitable to study the fluid flow around propellers with interaction from external bodies (Loureiro et al., 2021). In this method, the computational domain is split into two regions: rotating domains containing the propellers and stationary domains containing the flexible arms. The system is closed with a k-*ϵ* turbulent model with standard wall functions, which is especially suitable for flows involving rotation and recirculations.

**FIGURE 2:**
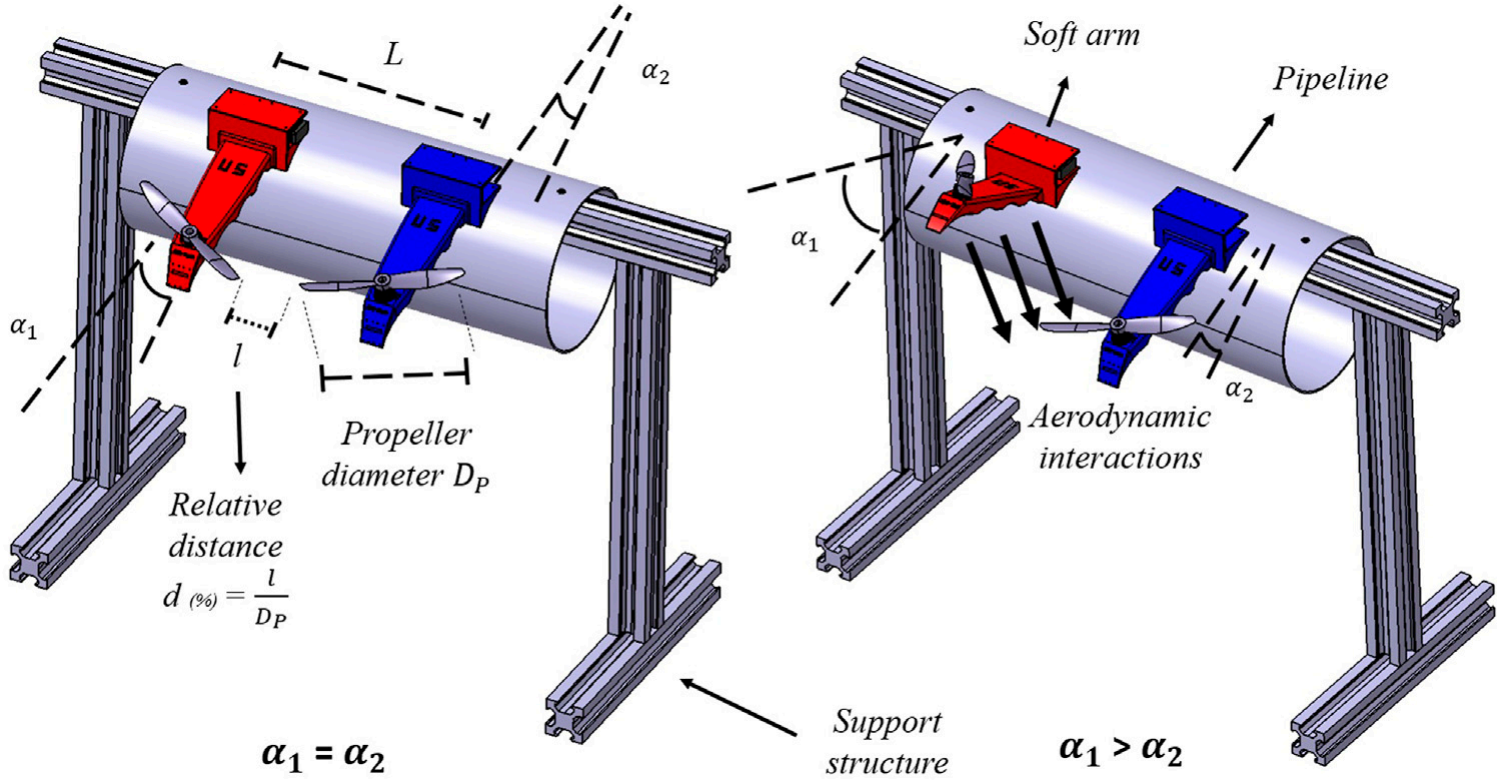
CAD model of the test bench used to analyze aerodynamic interferences between the arms and tuning of the PID controllers for hovering conditions.

**FIGURE 3:**
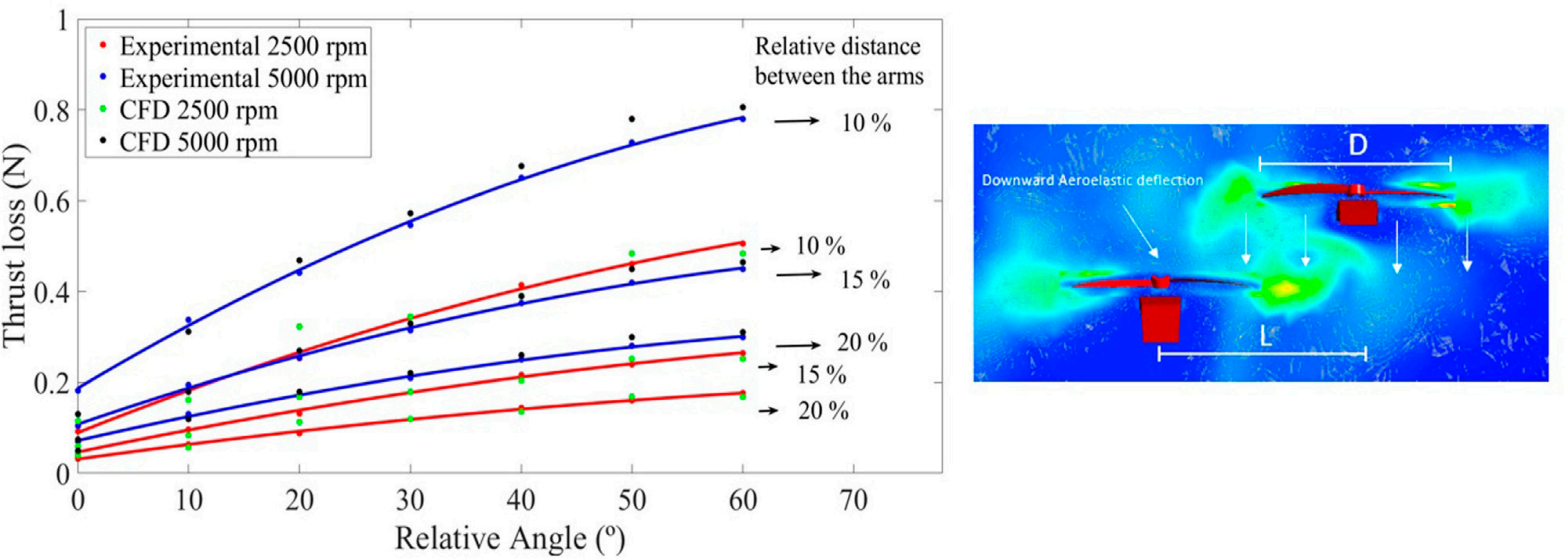
Left figure shows an analysis of the thrust loss in the lower arm due to aerodynamic interactions at 5000 rpm (blue curves) and 2500 rpm (red curves) by means of CFD (green and black dots) and experimental results (red and blue dots) for different distances and relative angles between the arms. In the right figure, pressure distributions in the vicinity of the arms are presented.

On the other hand, the experimental validation has been performed using the test bench shown in Figure 2, in which the base of the arms is fixed to the pipe and the interaction between parallel arms can be measured by varying the distance and the relative angle. The comparison is made in terms of the thrust loss by the propeller of the lower arm due to these interactions, and is shown in Figure 3.

Note that these steady-state interactions (rotor thrust inefficiencies) translate into variations in the deflection angle of the arms. Since the objective of this work is their stabilization (getting them all to remain at a similar deflection angle), having an understanding of the behavior of disturbances is essential for the design of the controller. Note that the magnitude of these aerodynamic disturbances does not depend on the properties of the material, while the deflections due to these interactions do.

$$\eta_{\mathrm{aero}}=\frac{T}{T_{\mathrm{ref}}}=1-\left(A+B\delta_{\alpha }+C\delta_{\alpha }^{2}\right){\left(\frac{2D_{P}}{2D_{P}+l}\right)}^{D}$$ (1)

Eq. 1 is proposed to analytically model such behavior, where *D*_*P*_ is the propeller diameter, *l* and *d* are the absolute and relative distance between the arms, respectively; while *δ*_*α*_ = *α*_1_ − *α*_2_. The values of the constants have been obtained by parametric adjustment from the average between the experimental and numerical data: *A* = 0.13, *B* = 0.014, *C* = −0.0002, and *D* = 1.22. Note that the influence of the rotational speed has not been included in the model due to its dimensionless character *η*_*aero*_.

### 3.2 Torsion and bending mechanical effects

**FIGURE 4:**
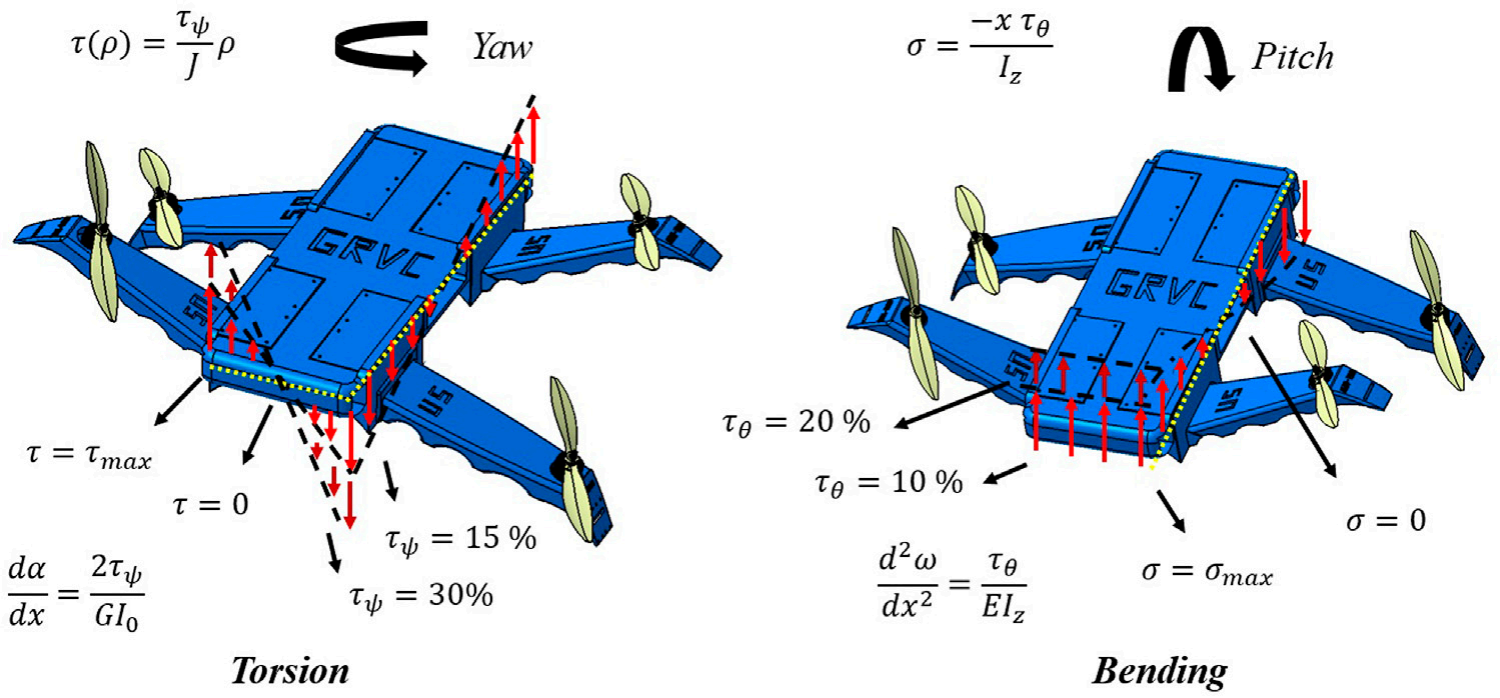
Illustration of the distribution of torsional (left) and bending (right) loads (during yaw and pitch/roll maneuvers, respectively) for different values of applied torque (*τ*_*ψ*_, *τ*_*ϕ*_, *τ*_*θ*_) from which the torsion angle *α*_*tor*_ and bending displacements (*ω*_*roll*_, *ω*_*pitch*_) are obtained.

**FIGURE 5:**
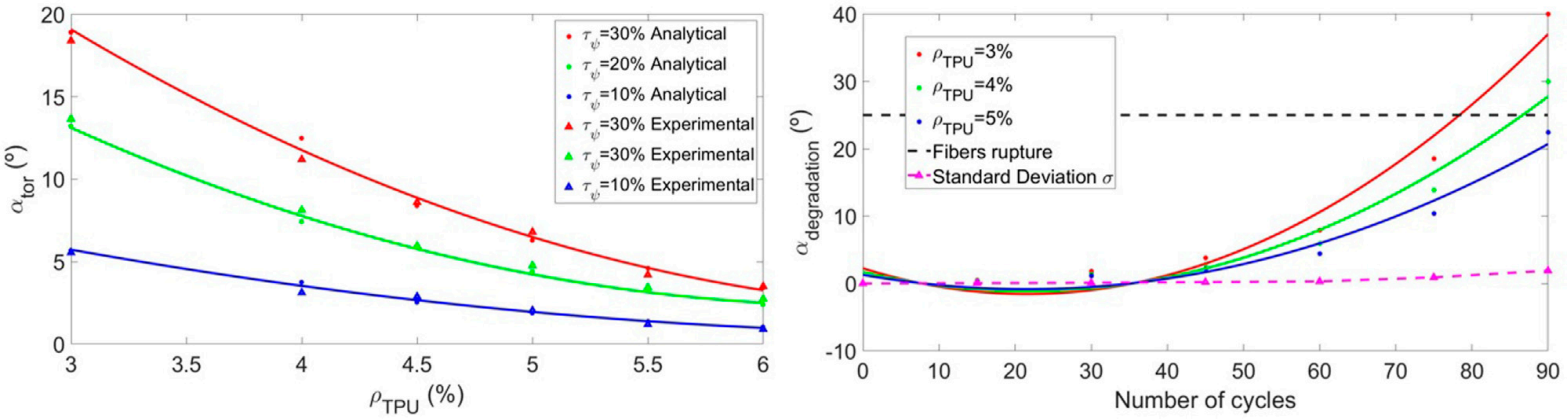
The left Figure shows the evolution of the torsion angle obtained analytically (dots) and experimentally (triangles) as a function of the infill rate *ρ*_*tpu*_. The right Figure analyzes arm deflections produced by material degradation as a function of the number of cycles *N*_*cycles*_. The magenta dashed line corresponds to the standard deviation *σ* of the experiments, while the limiting angle for fibers rupture corresponds to the black dashed line.

The elastic torsion of the UAV as a whole is a very important mechanical effect that must be considered to stabilize the UAV during the yaw maneuver. In this process (see Figure 4) an antisymmetric deflection occurs between the clockwise and counterclockwise arms, leading to a deformation of the central structure, which is also flexible, due to torsional loads. Considering this effect as a pure Saint-Venant torsion (due to large torsional inertia), Coulomb’s theory follows (2)

$$\tau \left(\rho \right)=\frac{\tau_{\psi }}{J}\rho$$ (2)

where *τ*(*ρ*) is the shear stress, *τ*_*ψ*_ is the torsional (*ψ* is the yaw angle) torque given by the autopilot, J is the torsional modulus of the material and *ρ* is the distance from the torsion axis to the point where the shear stress is measured. By applying the Lamé-Hooke and equivalence equations, the following expression is derived to estimate the displacements (torsion angle *α*) for given coordinates (3)

$$\frac{\partial \alpha }{\partial x}=\frac{2\tau_{\psi }}{GI_{0}}t$$ (3)

where *I*_0_ = *I*_*y*_ + *I*_*z*_ is the polar moment of inertia (the sum of the second moments of area) and G is the transverse modulus of elasticity (the relationship between G and J can be obtained analytically). Deflection angles *α*_*tor*_ induced by torsional loads can therefore be estimated and compared with experimental results. Figure 5 shows the evolution as a function of the infill rate *ρ*_*tpu*_ and the applied torque *τ*_*ψ*_.

$$\alpha_{\mathrm{tor}}=\left(A+B\rho_{\mathrm{tpu}}+C\rho_{\mathrm{tpu}}^{2}\right)\left(D\tau_{\psi }+E\tau_{\psi }^{2}\right)$$ (4)

The model (4) is proposed to characterize the torsion of the UAV (with the constants *A* = 53.83, *B* = −14.48, *C* = 1.02, *D* = 2.86, and *E* = 1.11), whose physical meaning can be interpreted in Figure 4. Note that as the flexibility of the material increases, the influence of torsion becomes more evident.

On the other hand, bending-type loads and deformations appear fundamentally during the roll and pitch maneuvers of the UAV (with respect to the main and secondary axes, x and y, in each case). The physical interpretation of these elastic effects can be observed in Figure 4, while an analytical approximation can be obtained using the Euler-Bernouille theory for roll (5) and pitch (6), respectively.

$$\sigma =\frac{y\tau_{\phi }}{I_{z}};\frac{\partial^{2}\omega }{\partial y^{2}}=\frac{\tau_{\phi }}{EI_{z}}$$ (5)

$$\sigma =-\frac{x\tau_{\theta }}{I_{z}};\frac{\partial^{2}\omega }{\partial x^{2}}=\frac{\tau_{\theta }}{EI_{z}}$$ (6)

where *σ* is the bending load, *I*_*z*_ is the inertia, *τ*_*ϕ*_ and *τ*_*θ*_ are the roll and pitch torques (*ϕ* and *θ* are the roll and pitch angles), E is Young’s modulus, and *ω* is the maximum bending displacement. The comparison between analytical results (deformations measurements in this case are complex to obtain experimentally) is shown in Tables 1 and the proposed models for roll (7) and pitch (8) adjust with great precision to them (with the constants *A* = 8.76, *B* = −1.2, *C* = 0.08, *D* = 2.92, and *E* = 0.91 for roll and *A* = 4.38, *B* = −0.78, *C* = 0.06, *D* = 2.34, and *E* = −0.43 for pitch).

$$\omega_{\mathrm{roll}}=A+\left(B\rho_{\mathrm{tpu}}+C\rho_{\mathrm{tpu}}^{2}\right)\left(D\tau_{\phi }+E\tau_{\phi }^{2}\right)$$ (7)

$$\omega_{\mathrm{pitch}}=A+\left(B\rho_{\mathrm{tpu}}+C\rho_{\mathrm{tpu}}^{2}\right)\left(D\tau_{\theta }+E\tau_{\theta }^{2}\right)$$ (8)

**TABLE 1:** Analysis of the maximum deformations (*ω*_*max*,*roll*_, *ω*_*max*,*pitch*_) in the flexible UAV due to bending loads for changes in the infill rate *ρ*_*tpu*_ and the applied torques *τ*_*ϕ*_ and *τ*_*θ*_

*ρ*_*tpu*_ (%)	*τ*_*ϕ*_/*τ*_*θ*_(%)	*ω*_*max*,*roll*_ (cm)	*ω*_*max*,*pitch*_ (cm)
4	15	2.41	0.75
5	15	2.18	0.67
6	15	1.93	0.63
4	30	5.08	1.47
5	30	4.55	1.31
6	30	4.25	1.23

### 3.3 Material degradation

This section is dedicated to the variability in the mechanical properties of the material, either due to imperfections during additive manufacturing, or due to material degradation after a certain number of deflection cycles of the arms. Unlike the previous ones, this mechanical effect has been analyzed from a purely experimental point of view.

The experiments have been carried out by applying the arm a series of load cycles and analyzing the stiffness loss (extra deflections observed for the same thrust). To guarantee the precision of the results, for each case (infill rate *ρ*_*tpu*_ and number of cycles *N*_*cycles*_) eight experiments have been carried out and the arithmetic mean has been taken.

The evolution of *α*_*degradation*_ is shown in Figure 5 and is defined analytically by the model (9), in which the constants have been fitted (*A* = 2.25, *B* = −0.35, *C* = 0.0082, *D* = 0.31, and *E* = −0.004). It is demonstrated how this effect is noticeable after 40–50 cycles, with the material being practically unusable after 100 cycles.

$$\alpha_{\mathrm{degradation}}=A+\left(BN_{\mathrm{cycles}}+CN_{\mathrm{cycles}}^{2}\right)\left(2-D\rho_{\mathrm{tpu}}+E\rho_{\mathrm{tpu}}^{2}\right)$$ (9)

Nevertheless, as material degradation is experienced regardless of the flight condition, it is more appropriate to unify the effects of material degradation and infill rate in a single relationship *ρ*_*tpu*_ = *ρ*_*tpu*_ (*N*_*cycles*_), obtained from the proposed model (9). In this way, material degradation is considered as a loss in the infill rate (10), where *A* = *ρ*_*tpu*,0_, *B* = −0.056 and *C* = −0.00092.

$$\rho_{\mathrm{tpu}}=A+BN_{\mathrm{cycles}}+CN_{\mathrm{cycles}}^{2}$$ (10)

## 4 Controller design

**FIGURE 6:**
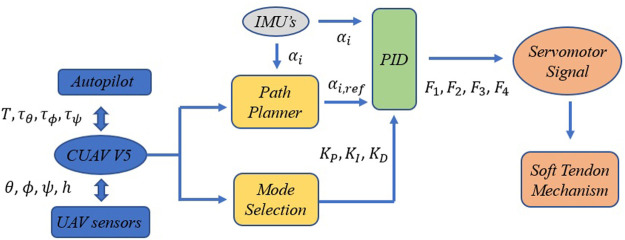
Descriptive diagram of the compensation system’s operating algorithm, including the path planner, mode selection and PID controller modules.

This section is dedicated to the analysis the system’s response to different aeroelastic disturbances, flight modes and material properties. These results are used to tune the gains of a case-adaptive PID controller using the Ziegler-Nichols method. The general layout of the controller is shown in Figure 6. The information related to the UAV sensors and the control signals generated by the autopilot are obtained from the CUAV V5 (*T*(*t*), *τ*_*ϕ*_(*t*), *τ*_*ψ*_(*t*), *τ*_*θ*_(*t*), *ϕ*(*t*), *ψ*(*t*), *θ*(*t*)). These data, together with the IMU data from each arm *α*_*IMU*_(*i*), is used to implement a control Algorithm 1 that performs the following actions:

• Path planner: calculation of the desired arm deflections *α*_*ref*_(*i*) = *δ*_*ref*_(*i*) + *ϕ* as a function of the flight condition. During hovering and yaw maneuvering, the ideal angle is *δ*_*ref*_(*i*) = 0, which means that the arms are parallel to the central body of the UAV *ϕ* (unless one of the arms has a smaller angle *δ*_min_ = min (*δ*_*i*_) < 0°, in which case that will be the reference deflection). During roll and pitch maneuvers, the desired inclination angles are selected (*ϕ*_*roll*_, *θ*_*pitch*_).
• Mode selection: is the module in charge of calculating the PID gains from analytical models, based on the main parameters that affect the disturbances (*T*(*t*), *τ*_*ϕ*_(*t*), *τ*_*ψ*_(*t*), *τ*_*θ*_(*t*), *ϕ*(*t*), *ψ*(*t*), *θ*(*t*)).
• PID controller: implementation of the PID controller from the modeled gains and IMU data. Then, the module sends the control signals to the servomotor and activates the soft tendon mechanism.

Among the heuristic methods for PID controller design, Ziegler–Nichols is one of the most robust for disturbance rejection. The multi-step tuning process starts by setting the integral and derivative gains to zero. Then, the proportional gain *K*_*p*_ is increased (from zero) until it reaches the ultimate gain *K*_*u*_, at which the output of the control loop has consistent oscillations with a period *T*_*u*_. Finally, the P, I, and D gains are set following these rules (*K*_*p*_ = 0.6*K*_*u*_, $K_{i}=1.2\frac{K_{u}}{T_{u}}$ , and *K*_*d*_ = 0.075*K*_*u*_ *T*_*u*_), a design that also aims to minimize overshoots.


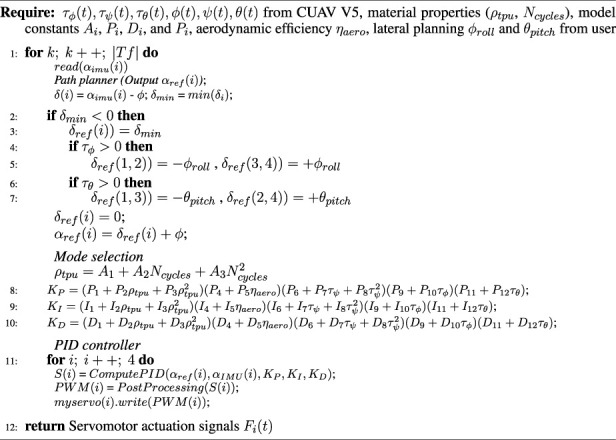

Algorithm 1 Path planner, mode selection and PID controllers for servomotor signals

### 4.1 Hovering

This section analyzes the disturbance response of the system during hovering of the soft vehicle. The main perturbations that play a relevant role are aerodynamic interferences (whose importance has been modeled with *η*_*aero*_) and changes in material properties or infill rate *ρ*_*tpu*_. The test bench used for the experiments is the one used in Section 3.1 (Figure 2), in which the arm is excited with a small thrust disturbance. Note that only the interaction between parallel arms is considered, since the influence of the arms on the opposite side is considered to be negligible.

**FIGURE 7:**
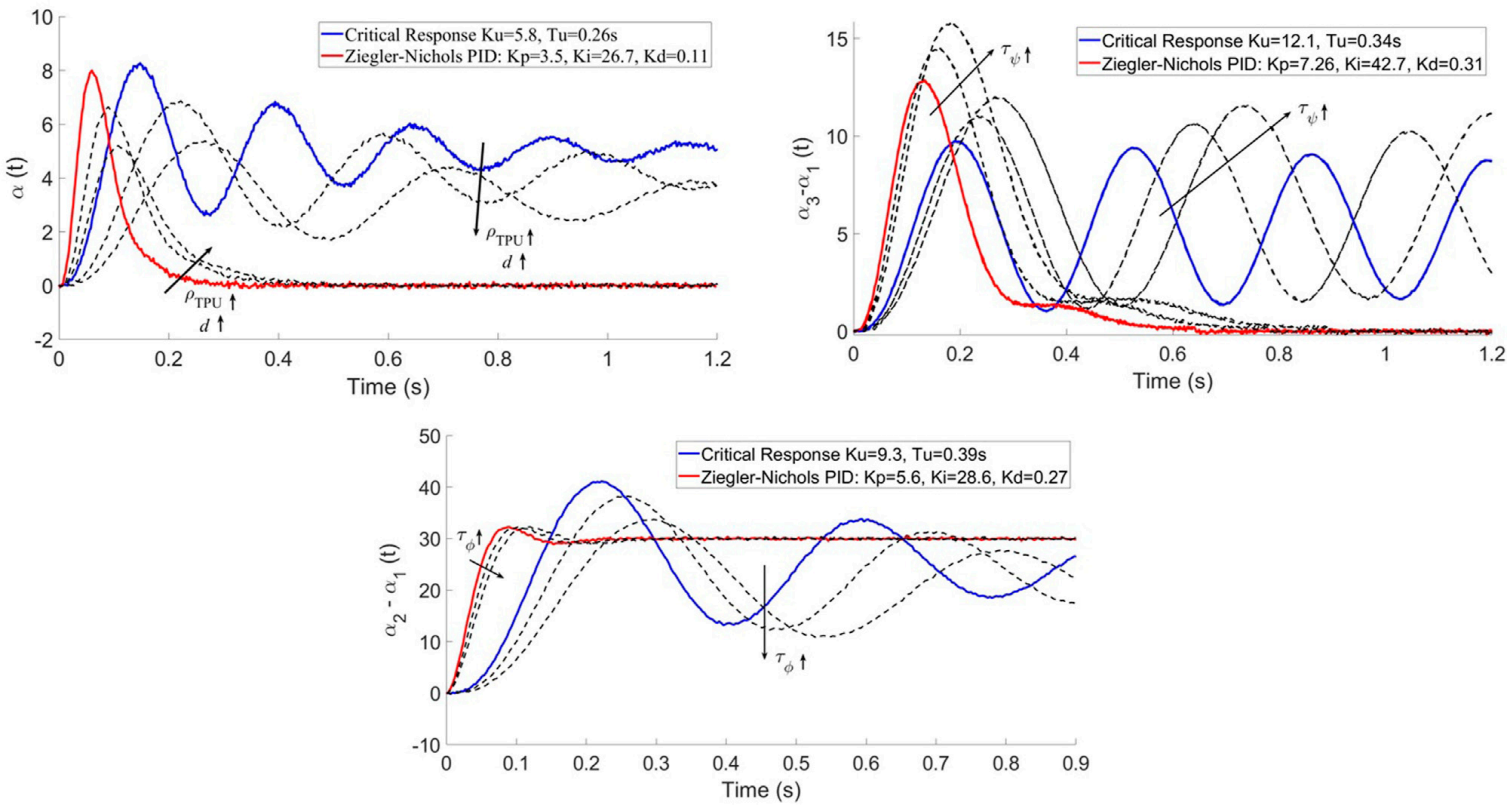
Disturbance rejection of the compensation system for hovering (top left), yaw (top right); and step response for pitch and roll (bottom). The blue curve shows the critical response of the system according to Ziegler-Nichols (*K*_*u*_, *T*_*u*_). The red curve shows the response of the system for the optimal tuning of *K*_*p*_, *K*_*i*_, and *K*_*d*_ according to Z-N method. The black dashed curves show the influence of the system parameters (*ρ*_*tpu*_, *η*_*aero*_, *τ*_*ϕ*_, *τ*_*ψ*_, *τ*_*θ*_) on the response.

Figure 7 shows the critical response (applying a gain *K*_*u*_ large enough to observe sustained oscillations) of the system for the case *ρ*_*tpu*_ = 4% and *d* = 15%. From this response (blue curve), which is completely determined by its critical period *T*_*u*_, the constants *K*_*p*_, *K*_*i*_, and *K*_*D*_ of the PID controller are calculated using the Ziegler-Nichols method.

It is observed that a decrease in the flexibility of the material (greater *ρ*_*tpu*_) leads to a decrease in the gain *K*_*u*_, and an increase in the oscillation period *T*_*u*_. This is because the system is slower due to stiffness. The same effects are observed when increasing the distance between the arms, since aerodynamic effects are damped.

### 4.2 Pitch *and* Roll

Pitch and roll maneuvers have the peculiarity that, in addition to aerodynamic disturbances and material degradation, they are also affected by mechanical bending of the UAV bending with respect to its primary and secondary axes (for pitch and roll, respectively). This causes the response of the system to be different and the PID controllers must be adjusted specifically. The experiments have been proposed as a deflection angle step response (from zero to *ϕ*_*roll*_). This does not prevent the controller design from being oriented towards disturbance rejection.

**FIGURE 8:**
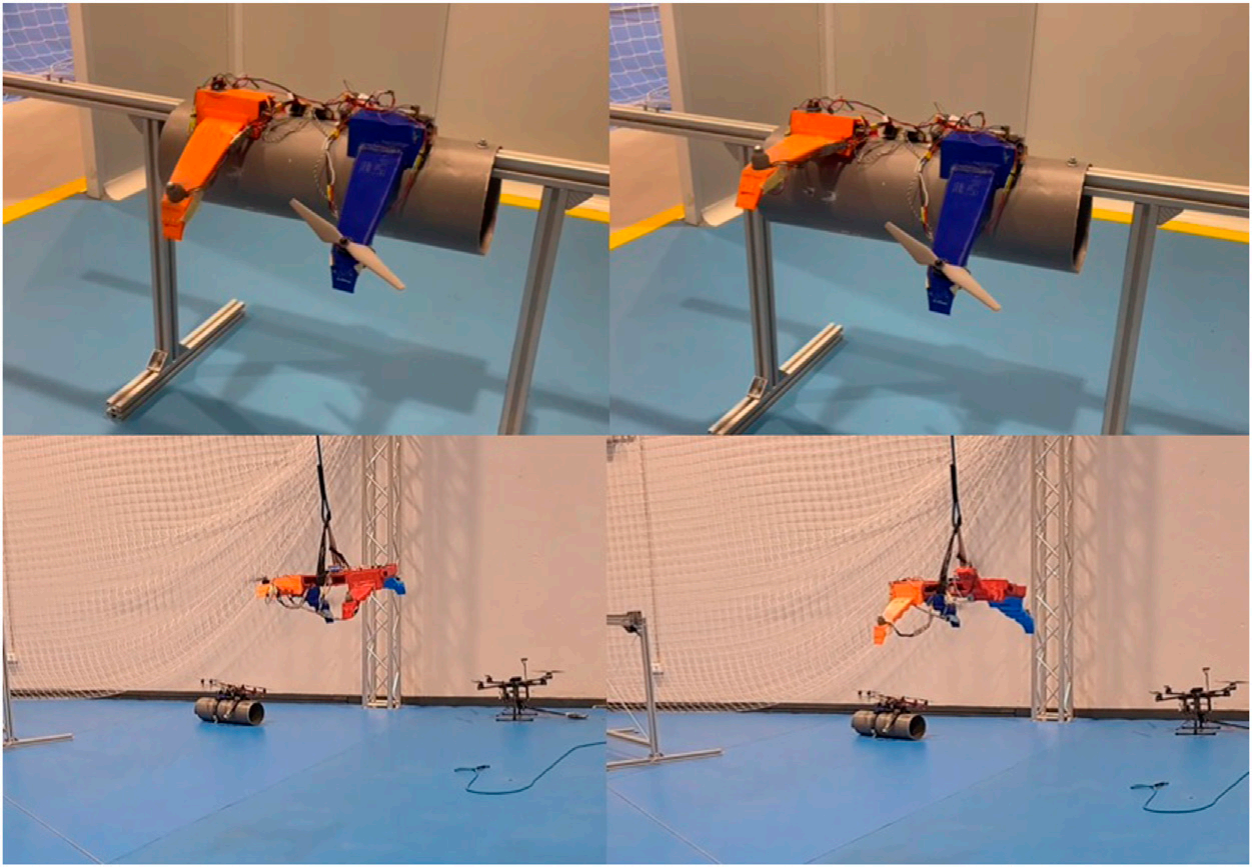
Prototypes used for the experiments. The upper images correspond to the test bench used for the analysis of the aerodynamic effects, while the bottom images correspond to the experiments of the soft drone during the tuning process using the Ziegler-Nichols method.

In this case, the test bench described in Figure 2 is not valid since the UAV must be analyzed as a whole so that the bending loads appear. However, performing the experiments in flight is complicated as well, since analyzing the limit response of the system according to Ziegler-Nichols implies destabilizing the UAV. Therefore, the UAV has been hung from a rope to facilitate the process (see Figure 8).

The results shown in Figure 7 demonstrate an increase in both the gain *K*_*u*_ and the critical period *T*_*u*_. The bending of the UAV forces to generate a greater control effort for the compensation, while the dynamics of the complete UAV is slower. A decrease in the applied torque *τ*_*ϕ*_ decreases the gain and the period. For the case *τ*_*ϕ*_ = 0, the hovering response is recovered. The results are shown for the case of roll but the same conclusions can be extrapolated to the pitch maneuver.

### 4.3 Yaw

This maneuver is particularly complex due to the effect of the torsional loads experienced by the UAV with respect to its main axis, as can be seen in Figure 5. In the same way as the roll maneuver, these mechanical effects and the aerodynamic interactions between the arms should be compensated by the PID controller. The experiment is in this case formulated in terms of disturbance rejection instead of step response.

*K*_*u*_ is greater than the previous maneuvers, which indicates that the control effort is maximum and the torsional loads are greater than those of bending. As shown in Figure 7 for the case *ρ*_*tpu*_ = 6% and *τ*_*ψ*_ = 15% The critical period is slightly lower with respect to the roll and pitch maneuvers, which shows that the yaw dynamics are slightly faster. The increase of *τ*_*ψ*_ causes an increase in the gain and insignificant changes in the critical period.

### 4.4 Adaptive PID controller

This section aims to design the adaptive PID controller that optimally selects gains for each flight condition, considering material properties and aeroelastic design. In order words, the influence of the system parameters (*ρ*_*tpu*_, *η*_*aero*_, *τ*_*ψ*_, *τ*_*ϕ*_ and *τ*_*θ*_) on the PID control actions.

The proposed model (11) brings together all the results obtained throughout this work in terms of analysis of aeroelastic disturbances and the system’s response to them. This model has been fitted with the constants shown in Table 2, obtained by interpolation from Figure 7. Note that a second-order fit has been chosen for the material properties *ρ*_*tpu*_ and for the yaw maneuver (torsion by *τ*_*ψ*_), since clear nonlinear behaviors have been observed in comparison with aerodynamic perturbations *η*_*aero*_ and bending during roll and pitch maneuvers (*τ*_*ϕ*_, *τ*_*θ*_).

**TABLE 2:** Adaptive PID controller model coefficients *A*_*i*_ for proportional, integral and derivative gains.

Control action	*A*_1_	*A*_2_	*A*_3_	*A*_4_	*A*_5_	*A*_6_	*A*_7_	*A*_8_	*A*_9_	*A*_10_	*A*_11_	*A*_12_
*P*	8.76	− 1.2	0.08	2.92	0.91	0.11	0.13	0.032	0.11	0.13	0.031	1.5
*I*	54.5	− 6.3	0.12	4.83	0.96	0.23	0.17	0.025	0.13	0.16	0.024	0.8
*D*	0.14	− 0.14	0.06	0.45	0.66	3.5	2.1	0.035	0.65	1.8	0.45	0.34

This model is introduced in Algorithm 1 and adjusts the optimal controller in real time for the tendon compensation system. Table 3 shows a summary of the model results for different parameters, comparing the quality of the controllers using the characteristic control times.

$$K_{\mathrm{adaptive}}=\left(A_{1}+A_{2}\rho_{\mathrm{tpu}}+A_{3}\rho_{\mathrm{tpu}}^{2}\right)\left(A_{4}+A_{5}\eta_{\mathrm{aero}}\right)\left(A_{6}+A_{7}\tau_{\psi }+A_{8}\tau_{\psi }^{2}\right)\left(A_{9}+A_{10}\tau_{\phi }\right)\left(A_{11}+A_{12}\tau_{\theta }\right)$$ (11)

**TABLE 3:** Compensation system PID response characterization (gains, stabilization times) for different material properties and flight modes.

*ρ*_*tpu*_ (%)	*η*_*aero*_	*τ*_*ψ*_(%)	*τ*_*ϕ*_(%)	*τ*_*θ*_(%)	*K*_*P*_	*K*_*I*_	*K*_*D*_	*T*_*I*_(*s*)	*T*_*D*_(*s*)
4	0.85	0	0	0	3.5	26.7	0.11	0.13	0.034
5	0.85	0	0	0	3.19	19.1	0.12	0.18	0.042
6	0.85	0	0	0	2.87	12.5	0.12	0.23	0.049
6	0.9	0	0	0	3.27	21.4	0.12	0.15	0.038
6	0.95	0	0	0	3.05	17.7	0.14	0.18	0.043
6	0.9	15	0	0	7.26	42.7	0.31	0.17	0.065
6	0.9	30	0	0	8.59	44.5	0.36	0.17	0.066
6	0.9	0	15	0	5.6	28.6	0.27	0.20	0.075
6	0.9	0	30	0	6.4	22.3	0.41	0.23	0.078
6	0.9	0	0	15	4.97	26.7	0.25	0.18	0.059
6	0.9	0	0	30	5.52	29.8	0.29	0.21	0.067

These results show that the reaction speed of the compensation system is greater in the yaw maneuver than in the roll and pitch maneuvers. On the contrary, the total stabilization time (for the complete elimination of the error in steady state) is smaller in the second case. It is also observed how the aerodynamic interactions notably increase the stabilization time of the system.

## 5 Results

### 5.1 Energetic analysis

This section aims to provide a summary of the results obtained by introducing the compensation system (CS) to stabilize the flexible vehicle, through a comparison of objective data. For this, the concept of energy efficiency is defined as

$$\eta =\frac{t_{CS}}{t_{\mathrm{ref}}}$$ (12)

calculated by measuring the average electrical consumption (*C*_*M*_) of the battery (4S of 1800mAh) in a 1-min period, for the specific flight condition evaluated. *t*_*CS*_ can therefore be calculated as the ratio between the battery capacity and the average consumption *C*_*M*_. It is greater the smaller the deflections of the arm with respect to the reference angle (therefore, the case without a compensation system (CS) is much less efficient). *t*_*ref*_ is the flight time of a vehicle with a similar design and weight, but with rigid arms (Ruiz et al., 2022b).

**FIGURE 10:**
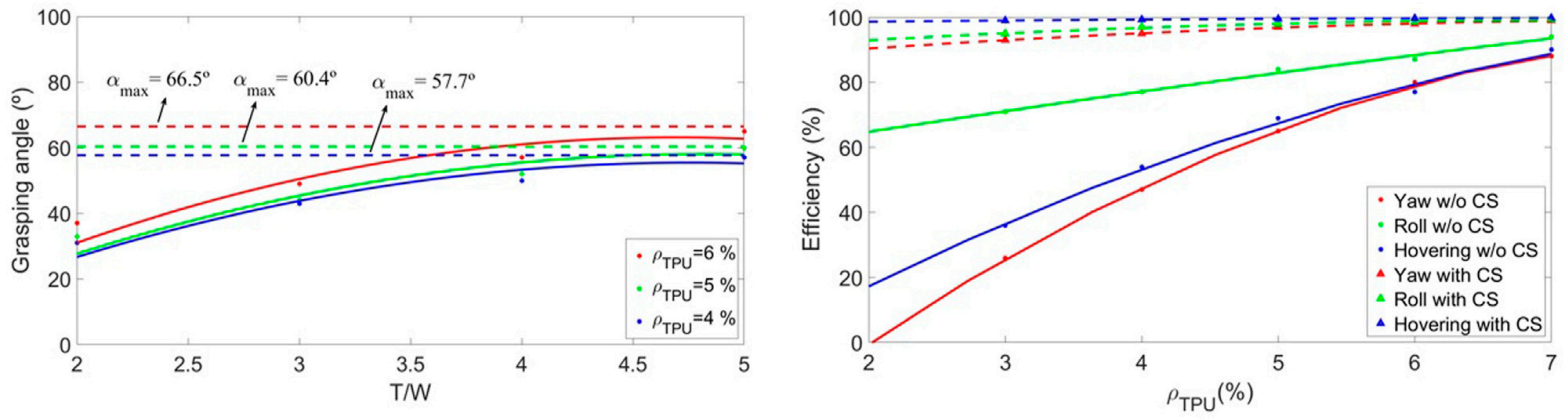
Left Figure shows the evolution of the efficiencies of the flexible UAV when introducing the compensation system CS (triangles) for hovering *η*_*hov*_ (blue curves), roll *η*_*ϕ*_ (green curves), and yaw *η*_*ψ*_ (red curves) maneuvers for different infill rates *ρ*_*tpu*_. Right Figure shows the evolution of the maximum grasping angles *α*_*grasp*,*max*_ of the flexible UAV for different infill rates *ρ*_*tpu*_ and thrust-to-weight ratios *T/W*.

The results (see Figure 10 and Table 4) show improvements in energy efficiency which are especially notable in the yaw maneuver (they reach 65% improvement for the highest degrees of flexibility). In the case of hovering, the performance of the compensation system is excellent (close to 100% efficiency) and therefore allows flying with lower densities. Finally, in the case of roll and pitch maneuvers, the impact on efficiency is not as significant, although it provides the UAV greater controllability.

**TABLE 4:** Comparison of the efficiencies of the flexible UAV when introducing the compensation system CS (triangles) for hovering *η*_*hov*_ (blue curves), roll *η*_*ϕ*_ (green curves), and yaw *η*_*ψ*_ (red curves) maneuvers.

*ρ*_*tpu*_ (%)	*η*_*ψ*_ *w/o CS*	*η*_*ψ*_ *w CS*	*η*_*ϕ*_ *w/o CS*	*η*_*ϕ*_ *w CS*	*η*_*hov*_ *w/o CS*	*η*_*hov*_ *w CS*
3	0.24	0.90	0.71	0.94	0.37	0.98
4	0.46	0.92	0.77	0.96	0.52	0.99
5	0.63	0.95	0.82	0.97	0.65	0.99
6	0.76	0.97	0.86	0.98	0.72	0.99
7	0.85	0.99	0.92	0.99	0.83	1

### 5.2 Application: Grasping maneuver limitations

**FIGURE 9:**
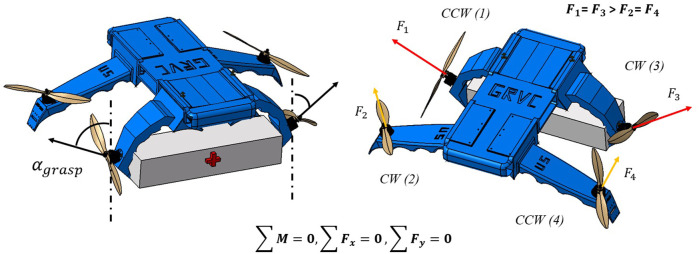
Description of the grasping concept with flexible arms in soft UAVs and definition of the maximum angle of grasping *α*_*grasp*,*max*_. This work intends to provide insight into the limitations of these angles in flight, while the actual grasping maneuver is not studied.

The grasping maneuver concept is shown in Figure 9. This paper intends to carry out a preliminary study on the possibilities of performing this maneuver in a soft UAV with four flexible arms/rotors using two of them. First, the balance of forces and moments shown in that Figure must be met (13, 14), leading to a relationship between the deflection angles of the arms that must be fulfilled for stable flight.

$$\sum M=0\to F_{1}+F_{4}=F_{2}+F_{3}\to F_{1}=F_{3}\geq F_{2}=F_{4}$$ (13)

$$\sum F=0\to \sin\left(\alpha_{\mathrm{grasp}}\right)F_{1}=\sin\left(\alpha_{A}\right)F_{2}\to \frac{\sin\left(\alpha_{\mathrm{grasp}}\right)}{\sin\left(\alpha_{A}\right)}=\frac{F_{2}}{F_{1}}\to \alpha_{A}=\sin^{-1}\left(\frac{F_{2}}{\sin\alpha_{\mathrm{grasp}}F_{1}}\right)$$ (14)

Then, this section calculates the maximum critical arm deflections *α*_*grasp*,*max*_ that can be achieved in flight as a function of the thrust-to-weight ratio of the UAV. Figure 10 analyzes this performance, throwing maximum deflection angles between 57 and 66° depending on the material.

The conclusion is that the maneuver is possible for maximum angles around 60%; although it is highly inefficient and requires oversizing the propulsion system of the aerial vehicle, due to working with such high thrust-to-weight ratios. Table 5 shows the numerical results of the efficiencies, calculated using the aforementioned methodology. Indeed, for the cases in which the angle is sufficient high to perform grasping (greater than 50–60°) the efficiencies are below 50%, so the maneuver is not feasible in these conditions.

**TABLE 5:** Efficiency comparison of the flexible UAV *η*_*grasp*_ during the grasping maneuver for different infill rates *ρ*_*tpu*_ and thrust-to-weight ratios *T/W*.

*ρ*_*tpu*_ (%)	*T/W*	*α*_*grasp*,*max*_ (°)	*α*_*A*_ (°)	*η*_*grasp*_
4	2	23.1	7.5	0.81
4	3	44.2	14.4	0.67
4	4	49.5	16.6	0.64
4	5	57.2	21.3	0.48
6	2	36.5	12.9	0.75
6	3	51.3	19.8	0.62
6	4	59.2	23.1	0.48
6	5	66.4	26.5	0.37

## 6 Conclusion

In this paper, a novel compensation system for soft aerial vehicle stabilization has been proposed. The actuation system is soft since it is made up of tendons arranged on the underside of the arm, which are wound on a 3D-printed reel and actuated by a servomotor.

This work has modeled the aerodynamic interactions between the arms as a function of relative distance and angle, as well as bending perturbations during pitch and roll maneuvers and torsion disturbances during yaw maneuvers. The degradation process of the material has also been analyzed in depth as a function of the number of cycles.

These studies have been the basis for the design of an adaptive PID that focuses on disturbance rejection, and whose gains are tuned according to variations in the system parameters and disturbances, using the Ziegler-Nichols method. The algorithms used are not very expensive from the computational point of view and their operation has been demonstrated in flight.

This system has made it possible to improve the energy efficiency of the system by up to 30%, depending on the flight conditions and the properties of the material. In turn, it has been possible to validate the flyability of the soft UAV at lower densities than the previous prototype, reaching an infill rate of 4%.

Finally, the conditions that must be fulfilled to perform the grasping maneuver have been analyzed, starting from the equilibrium of forces, and ending up with the maximum critical arm deflections that can be achieved in flight as a function of the thrust-to-weight ratio of the UAV. The conclusion is that the maneuver is possible for maximum angles around 60%. However, it is highly inefficient and requires oversizing the propulsion system of the aerial vehicle.

To deal with the aforementioned difficulties, several future alternatives are proposed: introducing extra arms (for example, an hexarotor in which two of the arms are dedicated to the task), the modification of the position of the propeller in the arm, closer to the central structure (although this has other negative consequences) or the implementation of a thrust vectoring system to guide the aerodynamic flow.

Another interesting research line would be to replace this external layer of compensation control with a complete control of the UAV based on artificial intelligence. Reinforcement learning techniques would be a suitable approach to deal with material degradation, unknown non-linear dynamics and time-varying parameters.

## Data Availability

The original contributions presented in the study are included in the article/supplementary material, further inquiries can be directed to the corresponding author.

## References

[B1] Ajanic E. Feroskhan M. Mintchev S. Noca F. Floreano D. (2020). Bio-inspired synergistic wing and tail morphing extends flight capabilities of drones. arXiv: Fluid Dynamics. 10.1126/scirobotics.abc2897 33115883

[B2] Augugliaro F. Lupashin S. Hamer M. Male C. Hehn M. Mueller M. W. (2014). The flight assembled architecture installation: Cooperative construction with flying machines. IEEE Control Syst. Mag. 34, 46–64. 10.1109/MCS.2014.2320359

[B3] Benosman M. (2016). “Chapter 2 - adaptive control: An overview,” in Learning-based adaptive control. Editor Benosman M. (Butterworth-Heinemann), 19–53. 10.1016/B978-0-12-803136-0.00002-6

[B4] Bernard M. Kondak K. Maza I. Ollero A. (2011). Autonomous transportation and deployment with aerial robots for search and rescue missions. J. Field Robot. 28, 914–931. 10.1002/rob.20401

[B5] Dilaveroğlu L. Özcan O. (2020). “Minicore: A miniature, foldable, collision resilient quadcopter,” in 2020 3rd IEEE International Conference on Soft Robotics (RoboSoft), New Haven, CT, USA, 15 May 2020 - 15 July 2020 (IEEE), 176–181. 10.1109/RoboSoft48309.2020.9115993

[B6] Dufour L. Owen K. Mintchev S. Floreano D. (2016). “A drone with insect-inspired folding wings,” in 2016 IEEE/RSJ International Conference on Intelligent Robots and Systems (IROS), 1576–1581. 10.1109/IROS.2016.7759255

[B7] Fishman J. Carlone L. (2020). “Control and trajectory optimization for soft aerial manipulation,” in 2021 IEEE Aerospace Conference (50100), Big Sky, MT, USA, 06-13 March 2021 (IEEE).

[B8] Garcia Rubiales F. J. Ramon Soria P. Arrue B. C. Ollero A. (2021). Soft-tentacle gripper for pipe crawling to inspect industrial facilities using uavs. Sensors 21, 4142. 10.3390/s21124142 34208723 PMC8235753

[B9] Gomez-Tamm A. E. Ramon-Soria P. Arrue B. C. Ollero A. (2019). Tcp muscle tensors: Theoretical analysis and potential applications in aerial robotic systems. Iberian Robotics conference.

[B10] Grzybowski J. Latos K. Czyba R. (2020). “Low-cost autonomous uav-based solutions to package delivery logistics,” in Advanced, contemporary control, 500–507.

[B11] Jimenez-Cano A. E. Martín J. Heredia G. Ollero A. Cano R. (2013). “Control of an aerial robot with multi-link arm for assembly tasks,” in 2013 IEEE International Conference on Robotics and Automation, 4916–4921.

[B12] Kim D. Kim S.-H. Kim T. Kang B. B. Lee M. Park W. (2021). Review of machine learning methods in soft robotics. PLOS ONE 16, e0246102–e0246124. 10.1371/journal.pone.0246102 33600496 PMC7891779

[B13] Kornatowski P. Mintchev S. Floreano D. (2017). “An origami-inspired cargo drone,” in 2017 IEEE/RSJ International Conference on Intelligent Robots and Systems (IROS), Vancouver, BC, Canada, 24-28 September 2017 (IEEE), 6855–6862. 10.1109/IROS.2017.8206607

[B14] Kovac M. (2016). Learning from nature how to land aerial robots. Science 352, 895–896. 10.1126/science.aaf6605 27199404

[B15] Leva A. (1997). Automatic tuning of pid regulators in presence of model perturbations near the desired closed-loop cutoff. Eur. J. Control 3, 150–161. 10.1016/S0947-3580(97)70073-6

[B16] Leva A. (2005). Autotuning process controller with improved load disturbance rejection. J. Process Control 15, 223–234. 10.1016/j.jprocont.2004.05.002

[B17] Loureiro E. V. Oliveira N. L. Hallak P. H. de Souza Bastos F. Rocha L. M. Delmonte R. G. P. (2021). Evaluation of low fidelity and cfd methods for the aerodynamic performance of a small propeller. Aerosp. Sci. Technol. 108, 106402. 10.1016/j.ast.2020.106402

[B18] Mintchev S. Shintake J. Floreano D. (2018). Bioinspired dual-stiffness origami. Sci. Robot. 3, eaau0275. 10.1126/scirobotics.aau0275 33141731

[B19] Nguyen D. Loianno G. Ho V. (2020). “Towards design of a deformable propeller for drone safety,” in 2020 3rd IEEE International Conference on Soft Robotics (RoboSoft), New Haven, CT, USA, 15 May 2020 - 15 July 2020 (IEEE), 464–469. 10.1109/RoboSoft48309.2020.9115983

[B20] Rao B. Gopi A. G. Maione R. (2016). The societal impact of commercial drones. Technol. Soc. 45, 83–90. 10.1016/j.techsoc.2016.02.009

[B21] Riviere V. Manecy A. Viollet S. (2018). Agile robotic fliers: A morphing-based approach. Soft Robot. 5, 541–553. 10.1089/soro.2017.0120 29846133 PMC6206552

[B22] Rowe W. B. (2009). “Chapter 11 - process control,” in Principles of modern grinding technology. Editor Rowe W. B. (Boston: William Andrew Publishing), 211–232. 10.1016/B978-0-8155-2018-4.50017-3

[B23] Ruggiero F. Lippiello V. Ollero A. (2018). Aerial manipulation: A literature review. IEEE Robot. Autom. Lett. 3, 1957–1964. 10.1109/LRA.2018.2808541

[B24] Ruiz F. Arrue B. Ollero A. (2022a). “A flexible propelled arm: Mechanical considerations for the use in uavs,” in International Conference on Unmanned Aircraft Systems (ICUAS). 10.48550/arXiv.2204.13987

[B25] Ruiz F. Arrue B. Ollero A. (2022b). “Sophie: Soft and flexible aerial vehicle for physical interaction with the environment,” in IEEE Robotics and Automation Letters. 10.48550/arXiv.2205.12883

[B26] Rus D. Tolley M. T. (2015). Design, fabrication and control of soft robots. Nature 521, 467–475. 10.1038/nature14543 26017446

[B27] Sahputro S. D. Fadilah F. Wicaksono N. A. Yusivar F. (2017). “Design and implementation of adaptive pid controller for speed control of dc motor,” in 2017 15th International Conference on Quality in Research (QiR) : International Symposium on Electrical and Computer Engineering, 179–183. 10.1109/QIR.2017.8168478

[B28] Sareh S. Siddall R. Alhinai T. Kovac M. (2017). Bio-inspired soft aerial robots: Adaptive morphology for high-performance flight. Biosyst. Biorobotics 17, 65–74. 10.1007/978-3-319-46460-2_9

[B29] Tognon M. Chávez H. A. T. Gasparin E. Sablé Q. Bicego D. Mallet A. (2019). A truly-redundant aerial manipulator system with application to push-and-slide inspection in industrial plants. IEEE Robot. Autom. Lett. 4, 1846–1851. 10.1109/LRA.2019.2895880

[B30] Xiang C. Guo J. Rossiter J. M. (2019). Soft-smart robotic end effectors with sensing, actuation, and gripping capabilities. Smart Mater. Struct. 28, 055034. 10.1088/1361-665x/ab1176

[B31] Yang D. Mishra S. Aukes D. M. Zhang W. (2019). “Design, planning, and control of an origami-inspired foldable quadrotor,” in 2019 American Control Conference (ACC), Philadelphia, PA, USA, 10-12 July 2019, 2551–2556. 10.23919/ACC.2019.8814351

[B32] Zhang K. Zhu Y. Lou C. Zheng P. Kovac M. (2019). “A design and fabrication approach for pneumatic soft robotic arms using 3d printed origami skeletons,” in 2019 2nd IEEE International Conference on Soft Robotics (RoboSoft), Seoul, Korea (South), 14-18 April 2019 (IEEE), 821–827. 10.1109/ROBOSOFT.2019.8722719

[B33] Zhao Z. Niu Y. Shen L. (2020). Adaptive level of autonomy for human-uavs collaborative surveillance using situated fuzzy cognitive maps. Chin. J. Aeronautics 33, 2835–2850. SI: Emerging Technologies of Unmanned Aerial Vehicles. 10.1016/j.cja.2020.03.031

[B34] Zheng Y. Du Y.-C. Su Z.-L. Ling H. Zhang M.-X. Chen S. (2021). Evolutionary human-uav cooperation for transmission network restoration. IEEE Trans. Ind. Inf. 17, 1648–1657. 10.1109/tii.2020.3003903

[B35] Zufferey R. Ancel A. O. Farinha A. Siddall R. Armanini S. F. Nasr M. (2019). Consecutive aquatic jump-gliding with water-reactive fuel. Sci. Robot. 4, eaax7330. 10.1126/scirobotics.aax7330 33137775

